# Supplementary material for: Reentrance of interface superconductivity in a high-Tc cuprate heterostructure
Source: Nat Commun. 2023 Nov 10;14:7290. doi: 10.1038/s41467-023-42903-1 (PMC10638369; doi:10.1038/s41467-023-42903-1)
Supplement: Supplementary file 1 — Supplementary Information [file 41467_2023_42903_MOESM1_ESM.pdf]

Reentrance of interface superconductivity in a high-T_c cuprate heterostructure

J.Y. Shen[1, 2, 3, 4†], C.Y. Shi[1, †], Z.M. Pan[3], L.L. Ju[1], M.D. Dong[1, 2, 3, 4], G.F. Chen[1, 2, 3, 4], Y.C. Zhang[1, 2, 3, 4], J.K. Yuan[3], C.J. Wu[3, 4, 5, 6, 7], Y.W. Xie[1], J. Wu[2, 3, 4*]

[1]*School of Physics, Zhejiang University, Hangzhou 310027, China*

[2]*Research Center for Industries of the Future, Westlake University, Hangzhou310024, China*

[3]*Department of Physics, School of Science, Westlake University, Hangzhou 310024, China*

[4]*Key Laboratory for Quantum Materials of Zhejiang Province, School of Science, Westlake University, Hangzhou, 310024, China*

[5]*New Cornerstone Science Laboratory, Department of Physics, School of Science, Westlake University, 310024, Hangzhou, China*

[6]*Institute for Theoretical Sciences, Westlake University, Hangzhou 310024, Zhejiang, China*

[7]*Institute of Natural Sciences, Westlake Institute for Advanced Study, Hangzhou 310024, Zhejiang, China*

[†]*These authors contributed equally to this work.*

[*]*Author to whom correspondence should be addressed: wujie@westlake.edu.cn*

Supplementary information

1. The x-ray diffraction (XRD) spectrums taken from the LSCO single layer and the LSCO/LCO bilayer

To characterize the crystal quality, we performed the XRD measurements

(Supplementary Fig. 1). The XRD peaks corresponding to the films verify both the single layer and bilayer are single crystals. As the doping x increases, the peaks become broader, indicating the crystal quality gradually degrades at higher doping. Eventually, the LSCO layer becomes amorphous for $x > 1.1$. The c-axis lattice constant c of the LSCO single layers decreases continuously as x increases, consistent with the previous reports[21, 22]. c of the LSCO/LCO bilayers shows a similar trend as a function of x. In principle, two separate peaks corresponding to the LSCO and LCO layers respectively should be present for the bilayer. However, the difference in c between them is too small to be resolved in the XRD spectrums and the measured c value is an average.

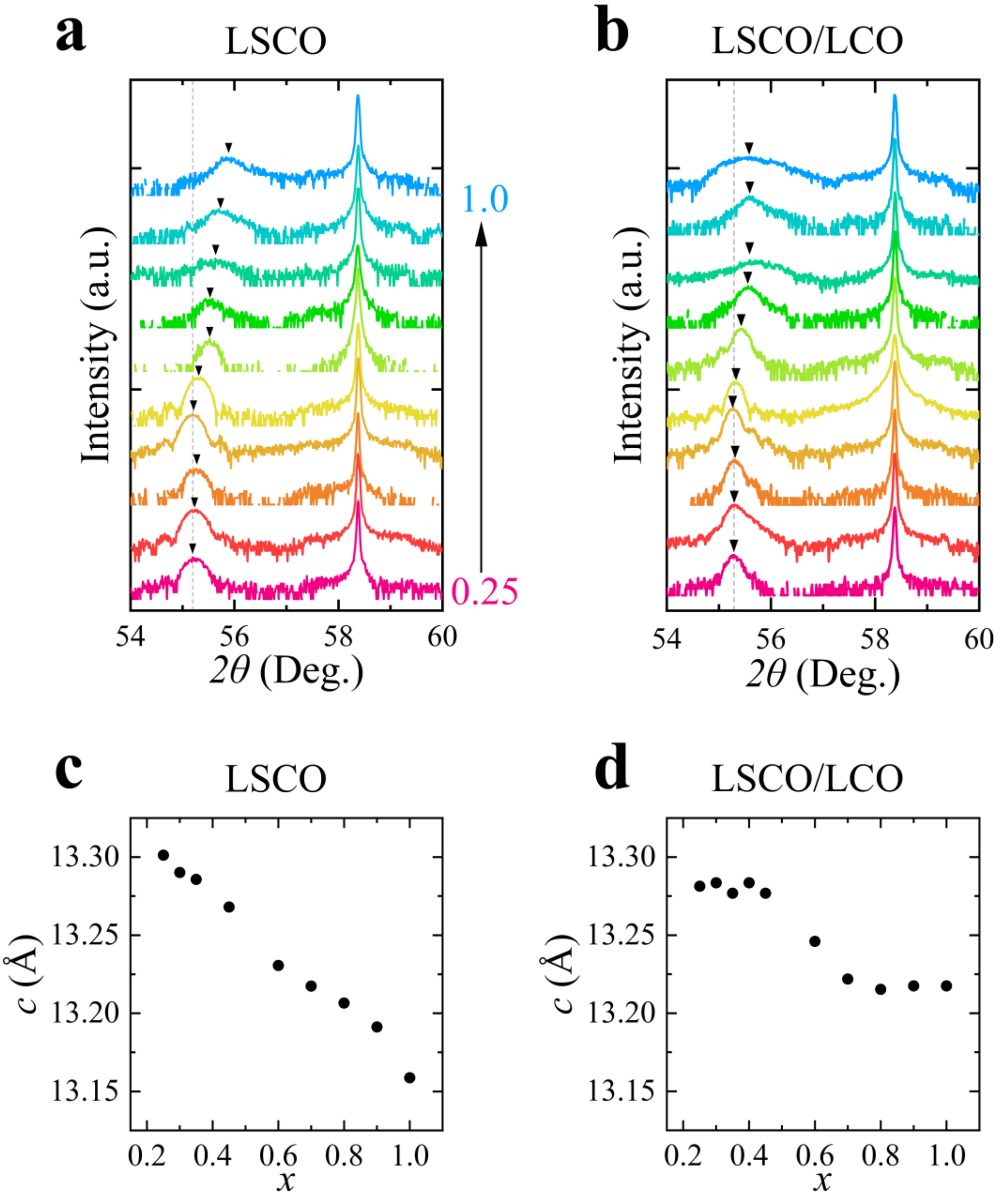

Supplementary Figure 1 | XRD spectrums from the LSCO single layers and the LSCO/LCO bilayers. The peak around 58.2° is from the $LaSrAlO_4$(001) substrate and the peak denoted by the arrow is from the LSCO (**a**) and LSCO/LCO bilayer (**b**). The lattice constant along the *c*-axis is measured as a function of the chemical doping x for the LSCO (**c**) single layers and LSCO/LCO bilayers (**d**).

To address the interfacial strain effect, we measured the in-plane lattice constants of LSCO/LCO bilayers. Apparently, the in-plane lattices of the epitaxially grown LSCO and LCO layers are identical to each other and both roughly follow the in-plane lattice of the substrate LSAO(001) at three representive dopings (Supplementary Table 1), which is expected under the conditions of epitaxial growth. The differences in a/b constant are within measurement uncertainty. Despite the drastic differences in T_c of the three dopings, the epitaxial tension remains approximately the same. Thus, it is safe to conclude that there are epitaxial strains exerted on LSCO/LCO bilayers but they are not the reasons for the reentrance of interface superconductivity.

	**a/b lattice constant (Å)**	**c lattice constant (Å)**
$LaSrAlO_4$ substrate	3.76	12.636
$La_{1.4}Sr_{0.6}CuO_4/La_2CuO_4$	3.77	13.246
$La_{1.2}Sr_{0.8}CuO_4/La_2CuO_4$	3.76	13.215
$LaSrCuO_4/La_2CuO_4$	3.75	13.217

Supplementary Table 1: The lattice constants of LSAO substrate and LSCO/LCO bilayers, determined by XRD measurements.

2. Evolution of the electric transport for the LSCO single layers ($0.45 \leq x \leq 1.0$)

For LSCO ($x = 0.45$), $\rho(T)$ shows a typical metallic behavior. At higher doping, $\rho(T)$ is non-monotonic and shows a minimum at a characteristic temperature T^*, below which the slope of $\rho(T)$ becomes negative (Supplementary Fig. 2a). As discussed in the main text, the weak localization behavior dominates in the range $T < T^*$. And T^* increases rapidly with x, implying the localization gets stronger at higher doping. For $x = 0.9$ and

1.0, $\rho(T)$ appears insulating for all temperatures that agrees well with the variable range hopping (VRH) expression under strong localization condition (Supplementary Fig. 2b).

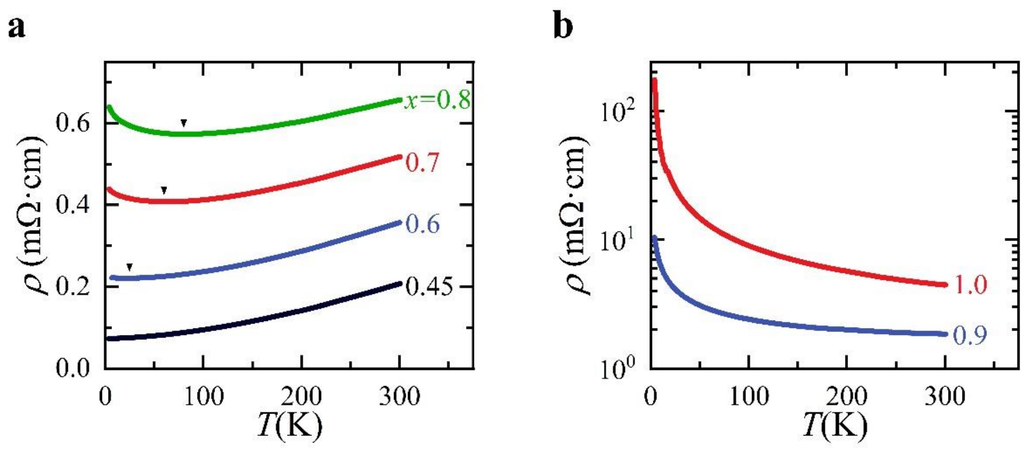

Supplementary Figure 2 | The longitudinal resistivity $\rho(T)$ for the LSCO single layers (0.45 ≤ x ≤ 1.0). The triangles mark the characteristic temperatures T^*, below which the localization behavior becomes significant.

3. Schrodinger-Poisson equation versus Poisson equation

Without localization effect, the charge transfer problem can be solved by the Poisson equation or Schrodinger-Poisson equation self-consistently. Here, we consider the 1D charge density distribution $p(z)$ along the z-direction near the LSCO/LCO interface. The density distribution can be solved from the Schrodinger equation for a given potential $\phi(z)$,

$$\left(-\frac{\hbar^2}{2m^*}\nabla^2 + q\phi(z)\right)\xi_i(z) = \varepsilon_i\xi_i(z). \quad (1)$$

ε_i is the ith energy level. In the numerical calculation, we would use the shooting method to impose the boundary condition $|\xi_i(-\infty)|^2 = x$ and $|\xi_i(\infty)|^2 = 0$. The hole density distribution $p(z)$ is given by summation of all the wavefunction density below the Fermi level. From $p(z)$, the electro-static potential $\phi(z)$ can be deduced based on the Poisson equation,

$$\nabla^2\phi(z) = -\frac{1}{\varepsilon}(p-n), \tag{2}$$

where ε is the dielectric constant and $n(z)$ is the background density distribution. The charge neutrality should be imposed as $\int_{-\infty}^{\infty} dz\big(p(z)-n(z)\big)=0$. The Schrodinger equation and Poisson equation should be solved self-consistently, and the resulting density distribution $p(z)$ is given in Supplementary Fig. 3a. As the original doping level x in the LSCO side increases, more and more holes are transferred into the LCO side. For comparison, the charge distribution obtained from solving the Poisson equation and equilibrium condition $e\phi(z)+\mu[p(z)] = \text{const.}$ is shown in Supplementary Fig. 3b. Here, the chemical potential depends on the hole density $p(z)$ linearly from Fermi liquid theory. The decreasing of hole density across the LSCO/LCO interface does not rely much on the detail of the chemical potential $\mu[p(z)]$. The comparison between two methods shows qualitative agreements.

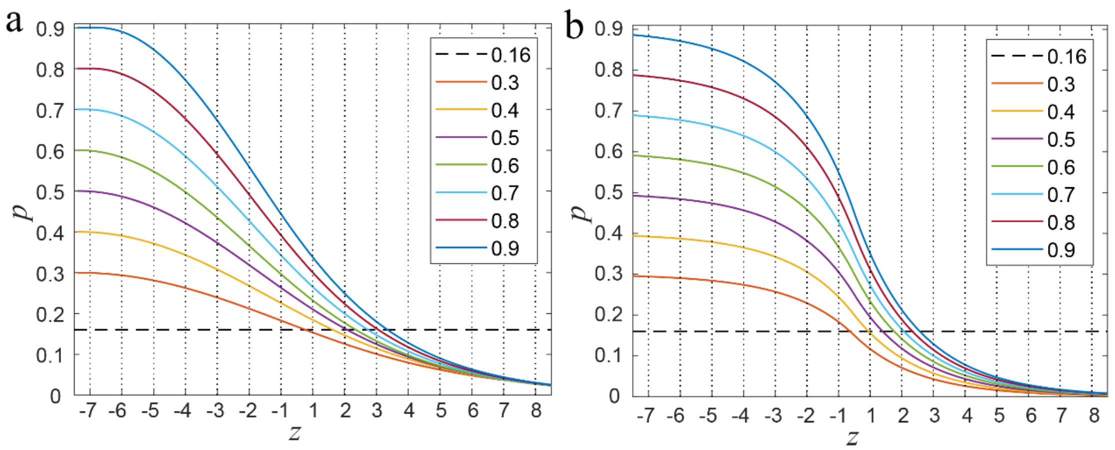

Supplementary Figure 3 | The calculated charge distribution from Schrodinger-Poisson equation (a) and Poisson equation (b) for LSCO/LCO ($0.3 \leq x \leq 0.9$) without taking into account the influence of charge localization. The value of z denotes the number of each CuO_2 plane counting from the interface. Positive z is on LCO side while negative z is on LSCO side. $p(z)$ deviates from $x(z)$ and the difference between them is the loss/gain of charge density due to charge transfer. The comparison between (**a**) and (**b**) shows there is no fundamental difference between the two approaches.

4. Localization-dominated charge redistribution

Interfacial charge transfer and consequently interface superconductivity, is dictated by the charge mobility in the LSCO layer for LSCO/LCO heterostructures. It changes dramatically in response to the crossover from weak localization ($x \leq 0.8$) to strong localization induced VRH ($x \geq 0.9$) in LSCO (Fig. S2). Thus, we shall discuss the two doping ranges separately. In the following, for simplicity we stick to the hole language, knowing that the hole and electron languages are ultimately equivalent in physics.

4.1 LSCO/LCO ($\mathbf{0.45 \leq x \leq 0.8}$)

For this doping range, as x increases in the LSCO layer, the disorder effect gets stronger and the density of pinning centers grows. Concomitantly, the amount of the localized charges increases at the cost of the mobile charges. The charge redistribution across the LSCO/LCO interface is dominated by the density of the mobile charges.

The charge density η transferred across the interface is determined by two counter-processes between the LSCO and LCO layers. One is the diffusion of mobile holes from the LSCO layer to the LCO layer, which is driven by the difference in the mobile hole densities p

$$J_{diff} = eD_L \frac{dp}{dz} = \mu_L k_B T \frac{dp}{dz}. \tag{3}$$

Here D_L and μ_L are the diffusion coefficient and the charge mobility in LSCO respectively, k_B is the Boltzmann constant, and $\vec{z}$ is the direction normal to the film. The diffusion current breaks the local charge neutrality and induces a built-in electric field E. From Gauss's law, the field E is induced by the transferred charge density η,

$$E = \frac{e}{\varepsilon a^2}\eta. \tag{4}$$

Here ε is the dielectric constant and a is the in-plane lattice constant. For simplicity, only the two layers between the interfaces are taken into account, which is sufficient if the interface charge transfer is smaller than 0.16.

Concomitantly, such an electric field drives a drift current for the mobile holes from the LCO layer to the LSCO layer

$$J_{drift} = ep\mu_R E, \tag{5}$$

where μ_R is the charge mobility in LCO.

The condition for equilibrium requires

$$J_{drift} = J_{diff}. \tag{6}$$

To solve the equations of (3)-(6), we retrieved $p(x)$ as well as other parameters from the $\rho(x, T)$ data shown in Supplementary Fig. 2 and use linear interpolation for intermediate doping levels. The calculated $\eta(x)$ and $T_c(x)$ are shown in Supplementary Fig. 4, which is in good agreement with experimental results in Fig. 1b. $\eta(x)$ is less than 0.16 and approaches zero at higher x since the mobile charges diminish with increasing amount of pinning centers. The results are not susceptible to the exact dependence of the mobile density on the doping level we assume. The interface superconducting temperature is determined by an empirical expression $T_c = 3200 \times (p - 0.06) \times (0.26 - p)$. Here p is the carrier density at the interfacial CuO_2 plane with the highest T_c. Please note that the maximum T_c of our bilayers is 32 K, lower than those synthesized by oxide-MBE due to rougher interface.

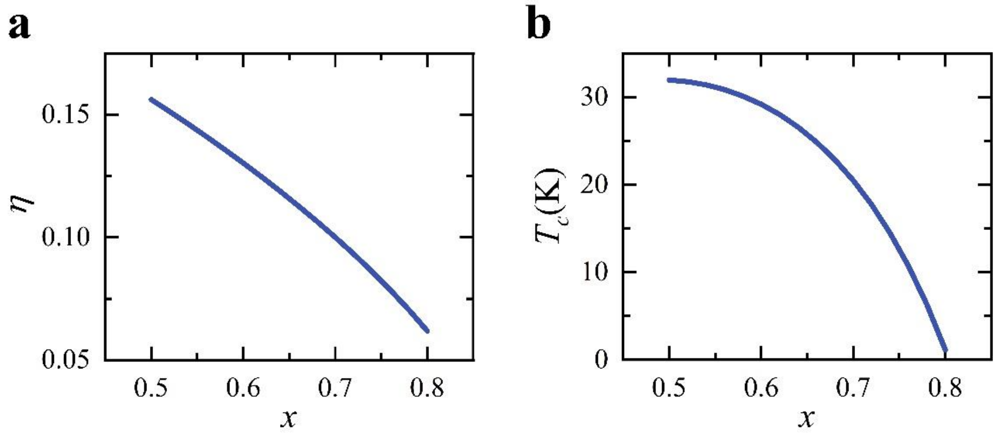

Supplementary Figure 4 | The calculated interfacial charge transfer η and the

resultant interface superconducting temperature T_c for LSCO/LCO bilayers (0.45 ≤ x ≤ 0.8).

4.2 LSCO/LCO ($0.8 < x \leq 1.0$)

In this high doping regime, all the charges in LSCO are localized, evidenced by the VRH behavior in transport. The charges can hop across the interface only when the depth of the potential well due to pinning is smaller than the chemical potential difference between LSCO and LCO. As a simple approximation, we use the Gaussian distribution for the distribution of the pinning potentials. Thus, the density of state for the localized charges in LSCO is

$$N(E') = \frac{N_0}{\sqrt{2\pi}\sigma} \exp\left(-\frac{(E'-u_0)^2}{2\sigma^2}\right). \tag{7}$$

Here we use u to denote the chemical potential to avoid confusion with the symbol μ for charge mobility. u_0 stands for the LSCO typical pining energy level due to the strong localization effect, which is smaller than the chemical potential. The energy is denoted by E', not to be confused with the electric field E. N_0 is a constant and σ is the spread of the distribution. Roughly speaking, the pinning energy is on the order of electron volt, which is comparable to the chemical potential difference at the LSCO/LCO interface.

After charges been transferred, the chemical potential of LSCO u_L is reduced and we have for the charge transfer

$$\eta = \int_{u_L-\Delta u}^{u_L} N(E')dE'. \tag{8}$$

Simultaneously, the chemical potential of LCO u_R increases by the amount of $\Delta\phi$ due to the built-in E field.

$$\Delta\phi \approx eE\frac{c}{2} \approx \frac{e^2 c}{2\varepsilon a^2}\eta. \tag{9}$$

The condition for equilibrium is the balance of the chemical potential, so

$$u_L - \Delta u = u_R + \Delta\phi. \tag{10}$$

Solving the equations (7)-(10) and treating N_0, σ, u_0 as fitting parameters, we obtained $\eta(x)$ and $T_c(x)$ (Supplementary Fig. 5) that reached a satisfactory agreement with experimental results in Fig. 1b.

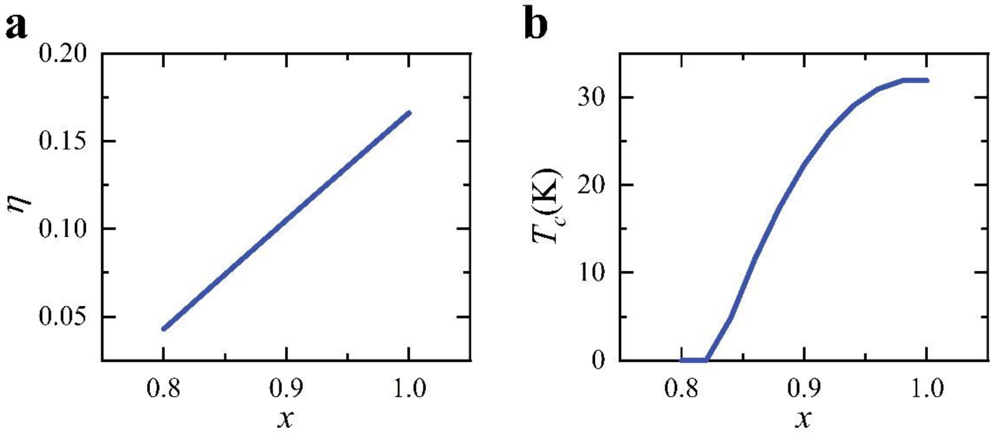

Supplementary Figure 5 | The calculated η and T_c for LSCO/LCO bilayers (0.8 < x ≤ 1.0).
